# Supplementary material for: Falling through the cracks: Modeling the formation of social category boundaries
Source: PLoS One. 2021 Mar 31;16(3):e0247562. doi: 10.1371/journal.pone.0247562 (PMC8011736; doi:10.1371/journal.pone.0247562)
Supplement: S1 Appendix — (PDF) [file pone.0247562.s001.pdf]

Appendices

A Similarity-based model

In the main text, we considered a model where differences between individuals are measured by the one-norm distance. This choice is driven by deriving a parsimonious model that captures the key behavior of the system and it is a common assumption in models that describe belief change from social influence (for example, in models based on variants of DeGroot learning [60]). However, research on multidimensional scaling and categorization suggests that a plausible model of how distance relates to categorization involves *similarity* [37]. Similarity describes how similar two attribute values are to the cognitive system, and is a nonlinear function of the distance. Here, we show that show our results of inbetweeners is not unique to the one-norm distance measure, but also arise with a measure of similarity.

Empirically, similarity between two positions x_1 and x_2 (denoted as $s(x_1,x_2)$) can be described as a nonlinear decreasing function with the distance between them in the form of

$$s(x_1,x_2) = \exp(-c|x_1 - x_2|) \ , \tag{A1}$$

where c is the sensitivity parameter—larger c means individuals are more sensitive to differences in attribute space. Variants of this functional form are used in many categorization and social judgment models [e.g., 18, 36].

Consider the group containing the left extreme of the attribute space. Similar to the derivation in the main text, the categorization error of an individual at position u, with group boundary z_1 is

$$\begin{aligned} err_1(u,z_1) \ &= \ \int_a^{z_1} [s(g_{\text{in}}(z_1), g_{\text{in}}(z_1)) - s(u,v)]^2 \rho(v) dv \\ &+ \int_{z_1}^{b} [s(g_{\text{in}}(z_1), g_{\text{out}}(z_1)) - s(u,v)]^2 \rho(v) dv \ . \end{aligned} \tag{A2}$$

The other equations of the model remain unchanged.

The model's results for uniform distribution is shown in Fig. A1, displaying solutions of boundary positions of the two groups (z_1^* and $z_2^* = 1 - z_1^*$) as a function of c . As c increases, meaning people more sensitive to differences in the attribute space, the social group boundaries become more exclusive. For all $c > 0$, the fixed point satisfies $z^* < 0.5$, meaning inbetweeners occur for all values of c.

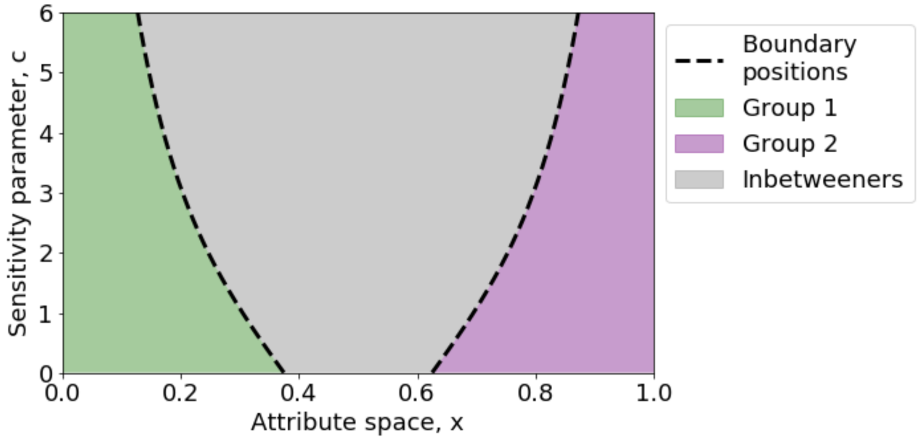

Figure A1. Stable fixed points of boundary positions for both groups, as a function of the sensitivity parameter, c. The dark grey region represents the inbetweeners. This result is for uniform attribute distribution $\rho(x)$ defined for x between 0 and 1.

B Individual-level model

In the main text, we used a group-level model in Section 2.2, where the group optimizes a collective in-group error. Here, we show an alternative formulation, where we prescribe the social process explicitly on the individual level.

In the group-level formulation, we consider all in-group individuals to form the same category boundary. In the individual formulation, we consider each individual (with position u) having a boundary position

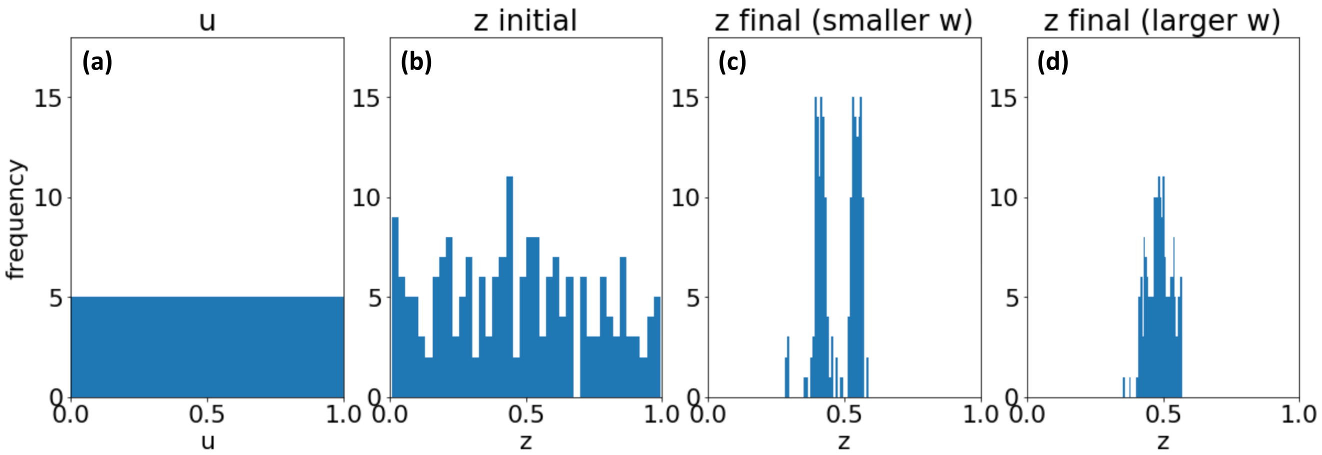

Figure A2. Simulations of the individual process level model for 200 individuals. (a) The population distribution in the simulation, an evenly spaced population. (b) The initial condition of boundary condition, drawn from a uniform distribution. (c) distribution of the individuals' boundaries after 500 time steps for $w = 0.2$. The distribution of the boundaries is bi-modal. (d) The distribution of the individuals' boundaries after 500 time steps for $w = 0.3$. The distribution is uni-modal.

($z(u)$), which can be distinct from that of other individuals. The part of the attribute space on the same side of the boundary is considered in-group for the individual, and the other side out-group. Note that here we no longer predict two group boundaries in attribute space like in the main text, but a distribution of boundary positions.

Each individual's utility consists of two components. One is the categorization accuracy, similar to Eq. 3, and the other is agreement with the boundary position of others in the same group. The categorization error for the individual at position u with boundary position z is,

$$err(u,z) = \sum_{v \in \text{ingroup}} (u-v)^2 + \sum_{v \notin \text{ingroup}} (|g_{\text{in}}(z) - g_{\text{out}}(z)| - |u,v|)^2 \tag{A3}$$

Equation A3 computes the individual categorization error the same way as Eq. 3 in the main text, but written in a discrete formulation, where the condition for v being in group is: (1) if $u > z$, $v > z$; and (2) if $u < z$, $v < z$. Otherwise, v is outgroup.

The cost (negative utility) function for each individual is,

$$f(u,z) = \frac{w}{N} err(u,z) + \frac{1-w}{N_{in}} \sum_{v \in \text{ingroup}} (z(u) - z(v))^2 \,. \tag{A4}$$

The first term is the error from categorization accuracy, and the second term is the cost from disagreeing with others in the in-group. The normalization term N is the total number of individuals in the system, and N_{in} is the number of people in the in-group. Parameter w, which is between 0 and 1, is the weight of the categorization accuracy compared to the need for having a boundary similar to others of the in-group. The dynamics for each individual is

$$\frac{dz}{dt} = -\frac{\partial f(u,z)}{\partial z} \,. \tag{A5}$$

This model generates a distribution of boundary positions. We simulate the system in discrete time, and an example is shown in Fig. A2. With a uniform distribution in attribute space, for parameters w smaller than a threshold, the system evolves to have a bi-modal distributed boundary positions. In numerical simulations, the transition between uni-modal to bi-modal occurs between $w = 0.2$ and 0.35, while the precise critical w value fluctuates within this range with the initial condition of the simulation. Intuitively, the presence of a transition can be understood as the following. When $w = 1$, where individuals are not affected by others at all, each individual's boundary is at their preferred position, and the distribution has no bi-modal behavior. When $w = 0$, individuals care only about social agreement, and two peaks would form on the attribute space. Thus a transition between these two kinds of distributions occurs for some w between 0 and 1, and the bi-modal boundary distributions occur when agreeing with others becomes more important, which would be similar to the model in the main text (group's members must agree on one position).

We can estimate the boundaries of the two groups using the peaks of the distributions. In the bi-modal distribution (smaller w, suggesting social influence is more important) , we approximate the two group boundaries with the two peaks of the distribution, and the individuals between these two peaks become inbetweeners, consistent with our findings in the main text. However, when individuals are less motivated by social influence (larger w), the distribution becomes unimodal. With estimating group boundaries with the peaks of distribution, the two group boundaries collide, and we no longer have inbetweeners.

C American National Election Studies Data

The American National Election Studies data used in this paper is the cumulative data file of 1940-2016, May 31, 2018 version. It was downloaded from `https://electionstudies.org/project/anes-time-series-cumulative-data-file/` on Oct 4, 2018.

The phrasing of the thermometer questions is as follows: "We'd also like to get your feelings about some groups in American society. When I read the name of a group, we'd like you to rate it with what we call a feeling thermometer. Ratings between 50 and 100 degrees mean that you feel favorably and warm toward the group; ratings between 0 and 50 degrees mean that you don't feel favorably towards the group and that you don't care too much for that group. If you don't feel particularly warm or cold toward a group you would rate them at 50 degrees. If we come to a group you don't know much about, just tell me and we'll move on to the next one. Using the thermometer, how would you rate the following...?" The three groups used in our analysis are "Democrats," "Republicans," and "political independents."

D American National Election Studies Data

In the main text, we use political independents to approximate those who are in the middle of the liberal-conservative attribute space. In Fig. A3, we show normalized histograms of self-reported liberal-conservative identification Democrats, Republicans and independents from ANES data. Indeed, independents tend to identify as the middle of attribute space, compared by Democrats and Republicans.

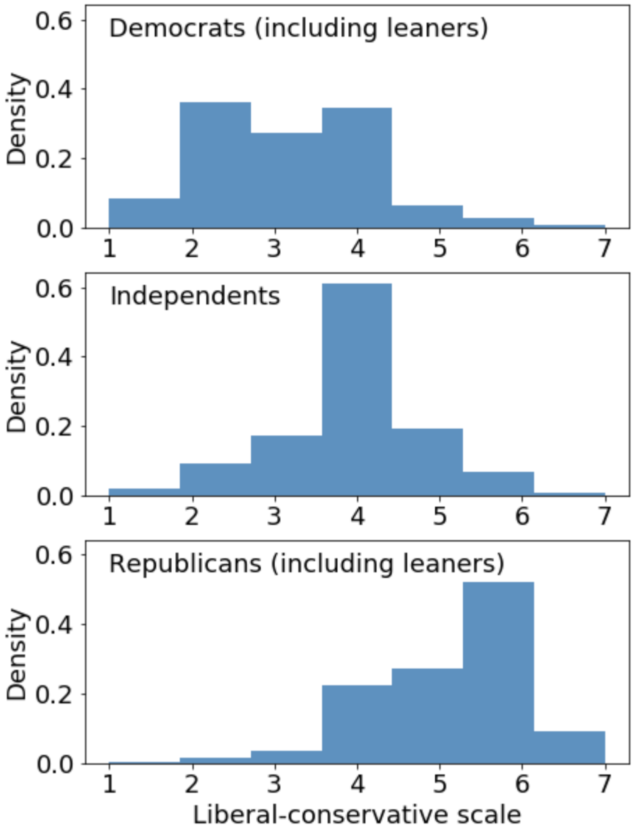

Figure A3. Self-identification on the liberal-conservative attribute space for Democrats, including leaners (top panel), Political Independents (middle panel), and Republicans, including leaners (bottom panel). Scale value 1 = extremely liberal, and 7 = extremely conservative.

References

60. DeGroot, M. H. Reaching a consensus. *J. Am. Stat. Assoc.* **69**, 118–121 (1974).
